# A novel mutation R190H in the AT-hook 1 domain of MeCP2 identified in an atypical Rett syndrome

**DOI:** 10.18632/oncotarget.18955

**Published:** 2017-07-28

**Authors:** Xiao Zhou, Yuangao Liao, Miaojing Xu, Zhong Ji, Yunqi Xu, Liang Zhou, Xiaoming Wei, Peiqian Hu, Peng Han, Fanghan Yang, Suyue Pan, Yafang Hu

**Affiliations:** ^1^ Department of Neurology, Nanfang Hospital, The Southern Medical University, Guangzhou, China; ^2^ Department of Psychiatry, Johns Hopkins University School of Medicine, Baltimore, MD, USA; ^3^ The First People’s Hospital of Chenzhou, Chenzhou, China; ^4^ Beijing Genomics Institute, Shenzhen, China; ^5^ Department of Radiology, Nanfang Hospital, The Southern Medical University, Guangzhou, China

**Keywords:** AT-hook 1, methylation of histone 3, MECP2, next generation sequencing technology, Rett syndrome

## Abstract

**Background:**

Mutations in *Methyl-CpG binding protein 2* (*MECP2*) have been identified as the disease-causing mutations in Rett Syndrome (RTT). However, no mutation in the AT-hook 1 domain of *MECP2* has been reported in RTT yet. The function of AT-hook 1 domain of *MECP2* has not been described either.

**Methods:**

The clinical and radiological features of a girl with progressive hyperactivity and loss of acquired linguistic and motor functions were presented. Next generation sequencing was used to screen the causative gene. Effect of the mutant protein on histone 3 methylation was assessed *in vitro* experiment.

**Results:**

The patient was diagnosed with an atypical RTT at the age of nine. Magnetic resonance imaging revealed a loss of whole-brain volume and abnormal myelination. Genetic analysis identified a de novo novel missense mutation of *MECP2* (NM_004992, c.570G->A, p.Arg190His). This mutation is located in the AT-hook 1 domain of MeCP2 protein. Overexpression of the mutant MeCP2 in cultured neuroblastoma cells SH-SY5Y revealed increased level of dimethylated histone 3 lysine 9, a transcriptional repressor marker.

**Conclusion:**

A novel missense mutation in AT-hook 1 domain of MeCP2 was identified in a patient with atypical RTT. Clinical data and *in vitro* experiment result imply that R190H mutation in AT-hook1 may cause dysfunction of MeCP2 and be a pathogenic variant.

## INTRODUCTION

Rett syndrome (RTT, OMIM #312750) is a devastating neurodevelopment disorder characterized by a period of apparently normal development followed by a progressive loss of acquired language and psychomotor skills. It mainly occurs in females with an incidence between 1/10,000 and 1/15,000 live births. According to the revised diagnosis criteria for RTT [1], RTT can be clinically diagnosed with a typical or atypical form. RTT is incurable up till now.

Mutations in the X-linked gene *Methyl-CpG binding protein 2 (MECP2)* are the primary cause for the vast majority of RTT cases [1–3]. Over 600 different genetic changes in the *MECP2* gene count for about 95-97% of typical RTT cases and 50-70% of atypical RTT cases. Approximately 95% *MECP2* mutations are de novo mutations [1, 4–6]. Even though *MECP2* mutations are neither necessary nor sufficient for clinical diagnosis of RTT [1], exploration of phenotype-genotype associations has offered clues for the mechanism study of MeCP2 in the role of RTT.

*MECP2* encodes two isoforms, MeCP2A (486 amino acids, aa) and MeCP2B (498 aa). The two isoforms differ in N-terminus by utilizing exon 2 or 1 respectively, but have same sequence by sharing both exon 3 and 4 [7, 8]. MeCP2A is expressed in all tissues, whereas MeCP2B is highly expressed in brain tissue and the expression increases during neuronal maturation [7, 9, 10]. MeCP2 contains two important functional domains: methyl-CpG binding domain (MBD) that selectively binds to methylated CpGs, and transcriptional repression domain (TRD) that interacts with various co-repressor complexes and regulates transcriptional activity of targeting genes [11, 12]. Recent evidence demonstrates that MeCP2 has a higher affinity to bind methylated CH (mCH, H representing nucleotide other than guanine) and hydroxymethylcytosine (hmC). Enrichment of mCH and hmC coincide with high expression of MeCP2 during postnatal neuronal maturation, which suggests that MeCP2 binding to mCH and hmC is important to modulate genes activities during neuronal maturation [13, 14]. In addition, MeCP2 contains three conserved AT-hook domains [15]: AT-hook 1 (aa 184-195), AT-hook 2 (aa 264-273) and AT-hook 3 (aa 341-364), of which AT-hook 2 can alter chromatin structures [15]. RTT patients carrying mutations with disrupted AT-hook 2 domain exhibited the most severe symptoms [15, 16]. Animal studies demonstrate that disruption of AT-hook 2 domain causes chromatin disorganization, a loss of chromatin remodeling protein ATRX (alpha thalassemia/mental retardation syndrome X-linked) from the heterochromatin, and mislocalization of ATRX within the nervous system. However, there is no mutation reported in the domain of AT-hook 1 or AT-hook 3 in RTT cases. Function of AT-hook 1 or AT-hook 3 domain remains unclear [15, 17].

In this study, we presented clinical features and cerebral structures of a late-onset atypical RTT, in which a de novo novel missense mutation R190H in the AT-hook 1 domain of MeCP2 has been identified by next-generation sequencing (NGS). When the mutant *MECP2* gene was overexpressed in the cultured SH-SY5Y cells, the level of dimethylated histone 3 lysine 9 (H3K9me2), a transcriptional repressor marker, was increased. Our results imply that missense mutation in *MECP2* (R190H) may disrupt AT-hook 1 function and cause clinic symptoms in the atypical RTT patient.

## RESULTS

### Clinical features of a chinese girl with atypical Rett syndrome

The patient is the second child of a healthy couple (mother: 32-year old; father: 34-year old). Her elder brother developed normally. She was born uneventfully at 41 weeks, weighing 3750 g. Her head circumference at birth was not available, and her status at birth was good without complaints of cyanosis, apnea, and convulsion or bleeding. Neonatal behavioral neurologic assessment was normal. She was able to raise her head at three months, sit at six months, start to speak at twelve months, and walk at 14 months. She could feed herself by using chopsticks and put on clothes by herself. By age of four, she was able to speak in complete sentences and recite long poems.

The patient developed normally until the age of five. Then she became hyperactivity and gradually lost acquired skills of language and fine motor function. Her electroencephalogram result showed diffuse slow activity, a prominent rhythmic delta activity, a loss of occipital-dominant rhythm while awake, and atypical sharp waves predominantly in the frontal, central and posterior regions during sleep. Genetic analysis was negative, which was revealed by multiplex ligation-dependent probe amplification (MLPA) tests for Prader-Willi syndrome, Angelman syndrome and RTT. Metabolism screening for deficiency of vitamin D, galactosemia, phenylketonuria, congenital hypothyroidism, and congenital adrenal hyperplasia was unremarkable. Her electrocardiogram was normal. Her disease progressed quickly. At age of 7, her cognitive functions deteriorated progressively and she developed gait abnormality. She had difficulty with descending stairs and feeding. She couldn’t recognize her parents and couldn’t speak or comprehend her native language. She was diagnosed with possible ADHD and global developmental delay by pediatricians in other hospitals. She took several-month medicine including L-carnitine, leucovorin, donepezil, or ganglioside separately but did not respond well.

She was 8-years-old when she came to our clinic center. In additional to the symptoms and signs mentioned above, she had abnormal muscle tone, diminished response to pain, constipation and occasional breath holding. Since her diagnosis was still unclear, we obtained her parent’s admission to conduct a genetic test for the patient to look for the potential disease-causing gene mutation by using NGS coupled with DNA target-capture array. Several gene variants including *MECP2* were identified and showed in the later text. Hinting by the genetic study, we closely observed her clinical features regarding to RTT in a follow-up study. Her head circumference was normal. She had some relatively preserved purposeful hand movements and nonverbal communication skills. She didn’t have stereotypic hand movements. By age 9, she became hyperactive and had difficulty falling sleep. She only slept 4 to 6 hours per day. The patient had a final diagnosis of atypical RTT when she clearly had regression period, met three out of the four main diagnosis criteria (loss of acquired purposeful hand skills, spoken language, and gait abnormality), and five out of eleven supportive criteria (breathing disturbances while awake, impaired sleep pattern, abnormal muscle tone, growth retardation, and diminished response to pain) [1].

The patient started to receive trazodone for the management of sleep and laxatives to treat constipation since 2014. Meanwhile, the patient went to a special school for rehabilitation of motor and language dysfunction. When she was 11 years old (Sept., 2016), her height was in a normal range (140 cm). Her symptoms became stable. Specifically, she could sleep well, walk by herself and picked small flowers and beans using her fingers.

### Imaging results

**Figure 1:**
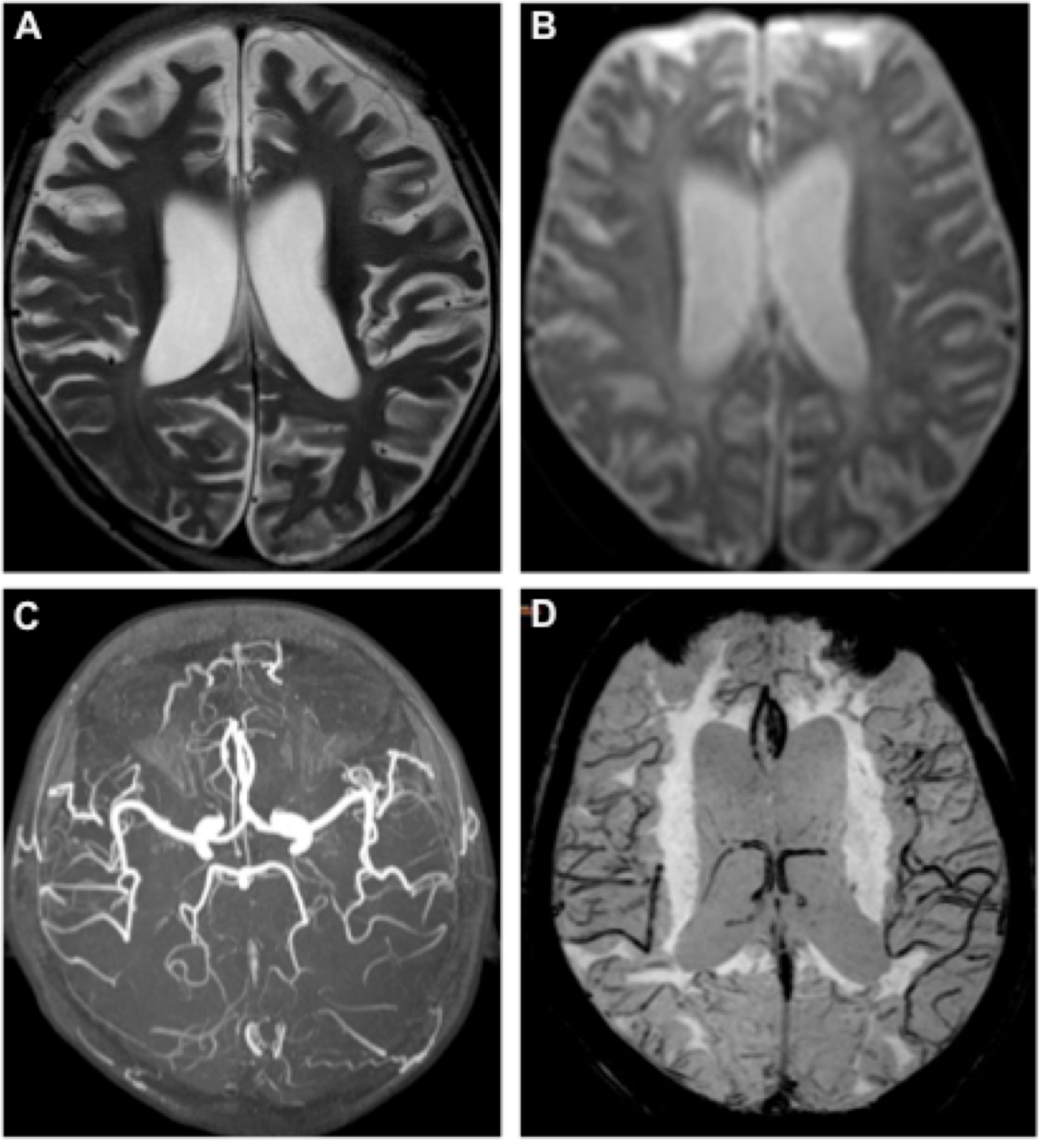
MR images of the RTT patient Axial T2-weighted image **(A)** showed diffuse high intensity areas in the posterior periventricular white matter without obviously worsen. DWI **(B)**, MRA **(C)**, and SWAN **(D)** at age of 8 were normal.

The patient’s brain MR images were showed in Figure 1. As in Figure 1A, the axial T2-weighted image indicated whole-brain atrophy and mild delayed non-progressive myelination characterized by diffuse high intensity areas in the posterior periventricular white matter. DWI (Figure 1B), MRA (Figure 1C) and SWAN (Figure 1D) revealed normal arteries and veins without ischemic or hemorrhagic lesions.

### Identification of a novel missense mutation (NM_004992, c.570G->A, p. Arg190His) in *MECP2* as the disease-causing gene mutation

To screen the disease-causing gene mutation for the girl, her DNA was analyzed by NGS coupled with DNA target-capture array including 1508 genetic diseases-causing genes according to Gene Reviews (NCBI) and published literature [18]. There were total six heterozygous variants found in the patient: *MECP2*, NM_001110792, c.605G->A, p.Arg190His, *DDOST*, NM_005216, c.59C->T, p.Pro20Leu, *HADHA*, NM_000182, c.1801_1802insG, *CACNA1H*, NM_021098, c.6517C->T, p.Pro2173Ser, *COL4A1*, NM_001845, c.3431C->G, p.Thr1144Arg, *GPR56,* c.1906C->T, p.Gln636Ter. All these variants occur in very low frequency in normal population compared in the Single Nucleotide Polymorphism Database (dbSNPS), international HapMap project database, one thousand project database, and local databases. These variants were not reported by others revealed by searching the Human Gene Mutation Database Data and PubMed.

**Figure 2:**
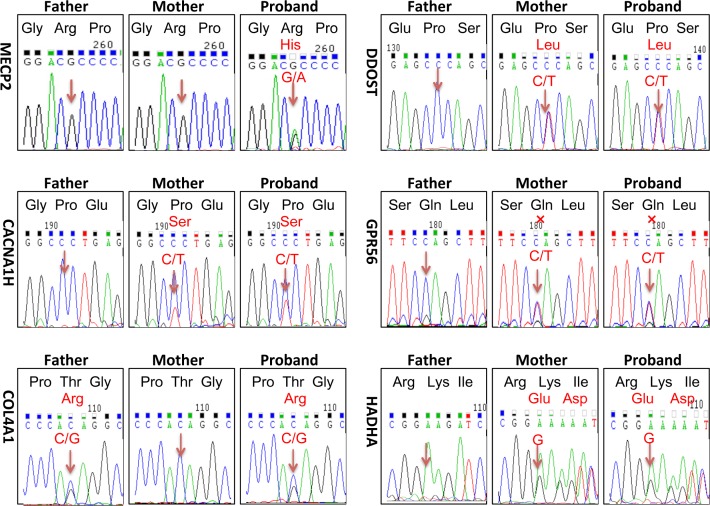
Sanger sequencing verified heterozygous variants identified by next generation sequencing technology There were total six heterozygous variants identified in the patient by NGS coupled with DNA target-capture array on Illumina HiSeq2000 platform: *MECP2*, NM_001110792, c.605G->A, p.Arg190His, *DDOST*, NM_005216, c.59C->T, p.Pro20Leu, *HADHA*, NM_000182, c.1801_1802insG, *CACNA1H*, NM_021098, c.6517C->T, p.Pro2173Ser, *COL4A1*, NM_001845, c.3431C->G, p.Thr1144Arg, *GPR56,* c.1906C->T, p.Gln636Ter. Sanger sequencing were taken to analyze each variant in the proband and her parents. Except for the de novo mutation of *MECP2* R190H, the proband inherited other five gene variants from her healthy mother or father. Mutated positions were point out by arrows. X represented termination.

NGS identified variants were further verified by Sanger sequencing in the patient and her parents (Figure 2). Except for the de novo variant *MECP2* R190H, the proband inherited other five gene variants from her healthy mother or father. Homozygous mutations in *DDOST, HADHA* or *GPR56* genes cause autosomal recessive diseases, thus, the heterozygous variants in these genes carried by the proband like their healthy parents were excluded for the pathogenic mutation. The patient inherited the *CACNA1H* variant from her mother. *CACNA1H* variants contribute to susceptibility to epilepsy [19]. Since neither the mother nor the patient had epilepsy, we excluded this variant from the pathogenesis list. Heterozygous mutations in *COL4A1* and *MECP2* cause autosomal dominant disorders. Protein function predicted by SIFT and Polyphen-2 software revealed probably damaging of the *COL4A1* or MECP2 variants. Mutations in *COL4A1* cause a wide spectrum of autosomal dominant disorders including porencephaly, brain small-vessel disease with hemorrhage, leukoencephalopathy, hereditary angiopathy with nephropathy, aneurysms and muscle cramp (HANAC) syndrome, and Walker–Warburg syndrome [20]. The patient inherited this variant from her healthy father and she did not exhibit symptom or abnormal imaging for the sign of *COL4A1* related disorders, indicated in Figure 1B-1D. Meanwhile, no lesion was found in her father’s images of MR and PET-CT scan (data not shown). Most *COL4A1* mutations are missense mutations on glycine positions. Because glycine residues are required for the formation of the core of the triple helix, non-glycine missense mutation is potentially less or no deleterious. The clinical relevance of this variant of *COL4A1* remains unclear. The significance of the *COL4A1* variant in the patient and the father will be closely followed up. Taken together, the phenotype of the atypical RTT patient matched to the genotype of mutation in *MECP2.* Thus, we consider the de novo mutation in *MECP2* as a probably disease-causing gene mutation for the patient. RettBASE has added the mutation to the database [6].

Since the patient’s MRI results indicated whole brain atrophy, we wanted to exclude other possible causative genes, which were not included in the array used. Further analysis by whole exome sequencing and CNV revealed negative results.

### Overexpression of mutant MeCP2 protein increased level of H3K9me2 in SH-SY5Y cells

**Figure 3:**
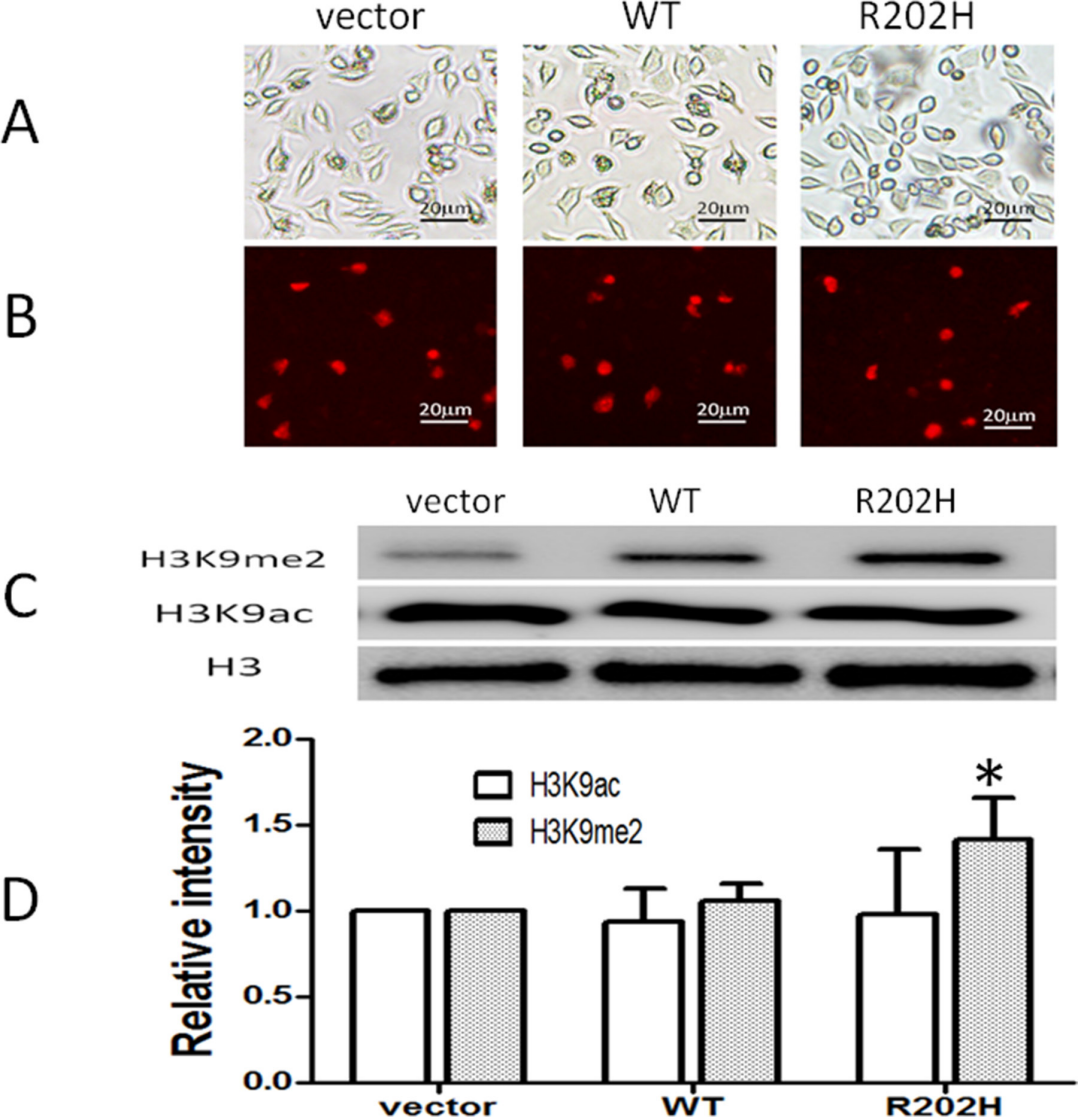
Missense mutation in the AT-hook 1 domain of MeCP2 increased level of H3K9me2 in SH-SY5Y cells SH-SY5Y cells were transfected by lipofectin 2000 reagent with plasmids: control DsRed-Monomer-N1, pDsRed-*MECP2B*, pDsRed-*MECP2B-R202H*. **(A)** Cells confluence pictures taken at x200 under fluorescence images microscope with white light. **(B)** Red signals represent transfected cells expressed DsRed protein or MeCP2B-Red fused proteins. **(C)** Western blot analysis: Level of H3K9me2 increased in cells transfected with pDsRed-*MECP2B-R202H* but not with vector control or wt *MECP2B*. The levels of H3K9ac were no difference among three groups. **(D)** H3K9me2 or H3K9ac /Histone 3 (H3) intensity ratio, *: P < 0.05, mutant group compared to vector or wt group. Data were from three independent experiments.

R190H mutation located in the AT-hook 1 domain of MeCP2. AT-hook 1 domain is extremely high conserved from fish to human and its function is unclear [15]. To check whether R190H mutation will affect the function of MeCP2, we constructed plasmids expressing wt MeCP2 or MeCP2B-R202H (related to R190H in MeCP2A). Because MeCP2 plays an important role in heterochromatin clustering through histone methylation and nucleosome remodeling [21], we assessed the effect of mutant MeCP2 on the modification of histone 3 proteins *in vitro* cultured cells. As shown in Figure 3A and Supplementary Figure 1, after transfected in SY-SH5Y cells (Figure 3B) and HEK293T cells (Supplementary Figure 1), mutant MeCP2 protein located in nuclear as well as wt MeCP2, indicated by the red fluorescence of fused proteins (confocal data further conformed, not shown here). Protein level of methylated H3 (H3K9me2) or acetylated H3 (H3K9ac) representing transcription repressor or activator respectively was assessed by Western blot assay. As indicated in Figure 3C&3D, H3K9me2 level significantly increased in SH-SY5Y cells transfected with mutant *MECP2*, but not in the vector control or wt *MECP2* transfected group (*p* < 0.05). Levels of H3K9ac were similar in all groups. Collectively, R190H mutation in the AT-hook1 domain of MeCP2 increased level of H3K9me2, which may result in transcriptional silence in neuronal cells.

## DISCUSSION

In this report, we described an atypical RTT patient with late-onset symptoms. Genetic analysis identified a de novo novel missense mutation in *MECP2* gene (NM_004992, c.570G->A, p. Arg190His). R190H mutation located in the AT-hook 1 domain of MeCP2 protein. Overexpression of mutant MeCP2 protein in SY-SH5Y cells leaded to increased level of H3K9me2, which is a transcriptional silence factor [22]. Our data suggest that disruption of AT-hook 1 domain by missense mutation of R190H may have epigenetic changes on neuronal cells and implications for the pathogenesis of the atypical RTT. This is the first case to report a missense mutation in the AT-hook 1 domain of MeCP2 in a late-onset atypical RTT patient. Other evidence to support that missense mutation in AT-hook 1 domain cause dysfunction of MeCP2 came from Bianciardi *et al.* study [17]. They reported two different missense mutations in or near AT-hook 1 domain lead to intellectual disability in two unrelated male patients.

The symptoms of our patient appeared later than most reported RTT patients carrying mutations in other domain of MeCP2 [1, 2]. Also, the patient did not show stereotypic hand movements, which is one of the four main diagnosis criteria for typical RTT. The dominant symptom of the patient was anxiety-like behavior, which was also the first symptom attracted her parents’ attention. Her anxiety-like behavior was attenuated along with insomnia, after she received trazodone, a selective serotonin reuptake inhibitors. Anxiety is more commonly reported in RTT patients with R133C and R306C mutations [1]. In addition, the patient showed a loss of cerebellar and cerebral volume, as well as mild abnormal myelination. Several studies find that gray-matter volume in the prefrontal, posterior-frontal, and anterior-temporal regions decreased significantly in RTT, whereas whole-brain white-matter volume was uniformly reduced [23–25]. At present, we do not have any explanation for the brain atrophy of the patient. To exclude other potential causative genes, exome sequencing and CNV analysis were further performed. No significant finding in the coding area of 2575 genes known to cause diseases and no large copy numbers difference or large deletions existed. However, we were unable to exclude additional causative genes that mutations are not in the coding area or at transcriptional levels or epigenetic levels.

To further access the pathological relevance of MeCP2 R190H, we compared wt or mutant MeCP2 on the histone 3 modification in cultured SY-SH5Y cells. We found that the level of H3K9me2 significantly increased by overexpressed mutant MeCP2 but not wt MeCP2. Shahhbazian M *et al* found that mice with truncated MeCP2 after the 308 amino residue display H3 but not H4 hyperacetylation [26]. However, they did not clarify the detail position of acetylation. Formation of H3K9me2 is catalyzed by euchromatic histone methyltransferases (HMTase), such as G9a [27] and G9a-related protein (GLP) [28]. G9a, DNMT3A and MeCP2 were found to form a larger repressive complex in an immature rodent model of ethanol-induced neurodegeneration. Pharmacological inhibition of G9a activity prior to ethanol treatment prevented H3 dimethylation and neurodegeneration [29]. Another study reveals that MeCP2 binds to and represses G9a, decreases H3K9me2 and increases expression of brain-derived neurotrophic factor in the central nucleus of the amygdala, which involving in regulation of emotional responses to pain and opioid reward [30]. These two studies demonstrate that MeCP2 plays as an H3K9me2 repressor. Thus, R190H mutation may cause dysfunction of MeCP2 and increased levels of H3K9me2, which may cause transcription silence and brain atrophy. These data imply that AT-hook 1 domain is critical to the function of MeCP2. The genetic study gives us a hint to the diagnosis, and our study is in line with the suggestion that *MECP2* mutations should be considered in people with neurodevelopmental disorders without the defined clinical features of RTT [31].

In conclusion, a novel missense mutation R190H in *MECP2* was identified in a patient with atypical RTT. Disruption of AT-hook1 domain of MeCP2 protein by R190H mutation increased the level of H3K9me2, which might cause transcription silence in cells. To elucidate the function role of AT-hook 1, future studies are undertaken in mutant mice carrying R190H mutation or AT-hook 1 domain mutation.

## MATERIALS AND METHODS

### Human subject

The ethics committee of the Nanfang Hospital, Southern Medical University, Guangzhou, China, approved this study. Written consent was obtained from the parents of the patient.

In Nov., 2012, an eight-year-old girl from the southern China area (Chinese Han) visited our clinic center due to progressively retardation and hyperactivity for three years. She was diagnosed with a possible attention-deficit/hyperactivity disorder (ADHD) and global development disorder at age of 7 in other hospitals. She was finally diagnosed with an atypical RTT at age of nine in our hospital based on diagnosis criteria for RTT [1].

### Magnetic resonance imaging acquisition

Magnetic resonance imaging (MRI) was taken with a 3.0 T magnetic resonance unit (Philips Medical Systems Philips, Achieva, the Netherlands), which consisted of T1-weighted images, T2-weighted images, fluid-attenuated inversion recovery images (FLAIR), diffusion weighted images (DWI), magnetic resonance angiography images (MRA), and susceptibility-weighted angiography (SWAN).

### Genetic test

All the genetic tests were performed by BGI (Shenzhen, China). The proband’s DNA sample was firstly performed with NGS coupled with DNA target-capture array on Illumina HiSeq2000 platform as reported [18]. Briefly, the solid phase array (Roche NimbleGen, Inc., Madison, WI, USA) was designed to capture all of the exons of 1508 genes involved in genetic diseases according to Gene Reviews (NCBI) and published literature [18]. Data analysis was performed by using the Illumina Bioinformatics analysis pipeline. Raw image files were then processed by Illumina basecalling Software 1.7 to base-call with default parameters and the sequences were generated as 90 bp pair end reads, which were subjected to alignment against human genome reference HG19 by BWA software (Burrows Wheeler Aligner). SNPs and indels were identified by SOAPsnp software and Samtools software. Variants were determined as novel or known variants according to dbSNP, HapMap databases, one thousand project database, 100 Chinese healthy adults, local data bases or from mutations previously reported in the literature [18], and the Human Gene Mutation Database at the Institute of Medical Genetics in Cardiff (HGMD) [18]. The potential deleterious effect was evaluated using Polymorphism Phenotypingv2 [32] and sorting intolerant from tolerant (SIFT) programs [33].

To check whether there might exist other causative genes which were not included in the array, whole exome sequencing and copy number variants (CNV) analysis were further applied by using Illumina HiSeq2500 platform. Briefly, the exome capture array was designed to capture all exons of the reported genes from Gene Reviews (NCBI) and published literature [18]. For exome sequencing, data analysis was similar as described above, except for the additional database including EXAC database, NHLBIGO Exome Sequencing Project (ESP). All the variants interpretation followed American College of Medical Genetics and Genomics Standards and guidelines for the interpretation of sequence variants-2015 (PMID: 25741868). For CNV analysis, firstly, the mean depth of each exon of the patient DNA sample was calculated, then divided by the mean depth of the sample, a normalized depth was resulted, named as G. Secondly, by the same method, normalized depth of other control samples were calculated and the mean normalized depth was achieved, named as H. According to statistical analysis of large number samples, the G/H value follows normal distribution, the thresholds of possible deletion or duplication were decided by different sample batches. All suspicious copy number variations will be confirmed by qPCR platform.

The following forward and reverse primers pairs were used for Sanger sequencing to further verify the selected variants in the patient and her parents: 5'-AGGAGGTCAGCCACATCA-3' and 5'-GGGG-CTCATCTTCTTCTTTC-3' for *CACNA1H* gene (exon 35), *5’*-GTGTGCTTGGGAGGGAACAG-3' and 5'-GCAGGGATGTGCAGTCTAGG-3' for *COL4A1*gene (exon40), 5'-GCGCCTTATCGCCAAAGCTG-3' and 5'-TCCCGCACGTTGAGGTTGTC-3' for *DDOST* gene (exon1), 5'-ACTCCCTGGTCAGCTACATC-3' and 5'-ATGACTGAGGCCCAGAGAAG-3' for *GPR56 (*exon14), 5'-CCCTGCATCGCTTCCTGTTC-3' and 5'-TTCTTCCACGAGGGCTTCTG-3' for *HADHA* gene (exon17), 5'-AGAGCGTTGTCACCACCATC-3' and 5'-TTTCACCTGCACACCCTCTG-3' for *MECP2* gene (exon 3). All PCR reactions were performed as following conditions: denaturation at 94°C for 5 min; 30 cycles of 94°C for 30s, 56°C for 30s, and 72°C for 35s; and a final extension at 72°C for 10 min. Sanger sequencing was performed with applied biosystems 3730 DNA analyzer.

### Assessment of histone 3 modification in cultured SH-SY5Y cells transfected with wide type or mutant *MECP2* gene

The open reading frame without stop codon of human *MECP2B* gene (NM_001110792) was synthesized and inserted in the EcoRI and ApaI cloning sites of plasmid pDsRed-Monomer- N1 (Takara Clontech, Mountain View, CA). The resulting plasmid was named as pDsRed- *MECP2B.* Mutagenesis of *MECP2B-R202H* (related to R190H mutation in the patient) was performed based on the template pDsRed-*MECP2B* according to the instructions of the KOD-Plus-Mutagenesis Kit (Toyobo, Tokyo, Japan). Mutation PCR primers were: forward 5’-ACCCCAAAGGGAGCGGCACCACGA-3’ and reverse 5’-GTCCCCGGCCTCTGCC- AGTTCCTG-3’. Both plasmids pDsRed-*MECP2B* (wide type, wt) and pDsRed-*MECP2B-R202H* (mutant) expressed MeCP2 proteins N-terminally fused to DsRed, a red fluorescence protein.

Neuroblastoma cell line SH-SY5Y was purchased from American Type Culture Collection (Manassas, VA). SH-SY5Y cells were cultured with DMEM containing 10% fetus bovine serum under 5%CO_2_ at 37°C. Plasmids containing vector control, wt or mutant *MECP2* were transfected in SH-SY5Y cells with lipofectamine 2000 reagents (Life Technologies Corporation, Grand Island, NY) according to the manufactory’s instruction. Transfected cells were cultured for 48 h and cell lysate were prepared. Cells were lysed in lysis buffer (PBS, pH 7.4, containing 1% Triton X-100, 0.1 mmol/l EDTA and complete proteinase inhibitor cocktail) (Roche, Basel, Switzerland). Histone proteins of cells were extracted with the EpiQuik™ Total Histone Kit (EpiGenTek, Farmingdale, NY).

Western blotting was performed routinely. Breifly, denatured protein samples were separated in 12% polyacrylamide gel electrophoresis and transferred to polyvinylidene fluoride membrane (Millipore, Billerica, MA). Membranes were incubated with one of the following primary antibodies: anti-histone 3 monoclonal antibody (mAb) or anti-acetylated-histone 3 (1:1000; Cell Signaling Technology, Danvers, MA), anti-dimethyl-histone H3 (Lys9) (1:1000; rabbit polyclonal Ab, upstate, NY), and anti-β-actin (1:5,000; CWBIO, Beijing, China). After incubated with anti-rabbit or anti-mouse horseradish peroxidase-conjugated secondary Abs (1:5,000; CWBIO), bands signals were visualized under the enhanced chemiluminescence scanner (Kodak Molecular imaging, Rochester, NY) and pictures were taken. The densities of protein bands were quantified by using ImageJ (National Institutes of Health, Bethesda, MD).

### Statistics

One-way ANOVA with Bonferroni’s post hoc tests was used via SPSS 21.0, and a *p* value of <0.05 was considered significant.

## SUPPLEMENTARY MATERIALS FIGURE


