# Supplementary material for: A novel mutation R190H in the AT-hook 1 domain of MeCP2 identified in an atypical Rett syndrome
Source: Oncotarget. 2017 Jul 28;8(47):82156–64. doi: 10.18632/oncotarget.18955 (PMC5669878; doi:10.18632/oncotarget.18955)
Supplement: Supplementary file 1 [file oncotarget-08-82156-s001.pdf]

A novel mutation R190H in the AT-hook 1 domain of MeCP2 identified in an atypical Rett syndrome

SUPPLEMENTARY MATERIALS

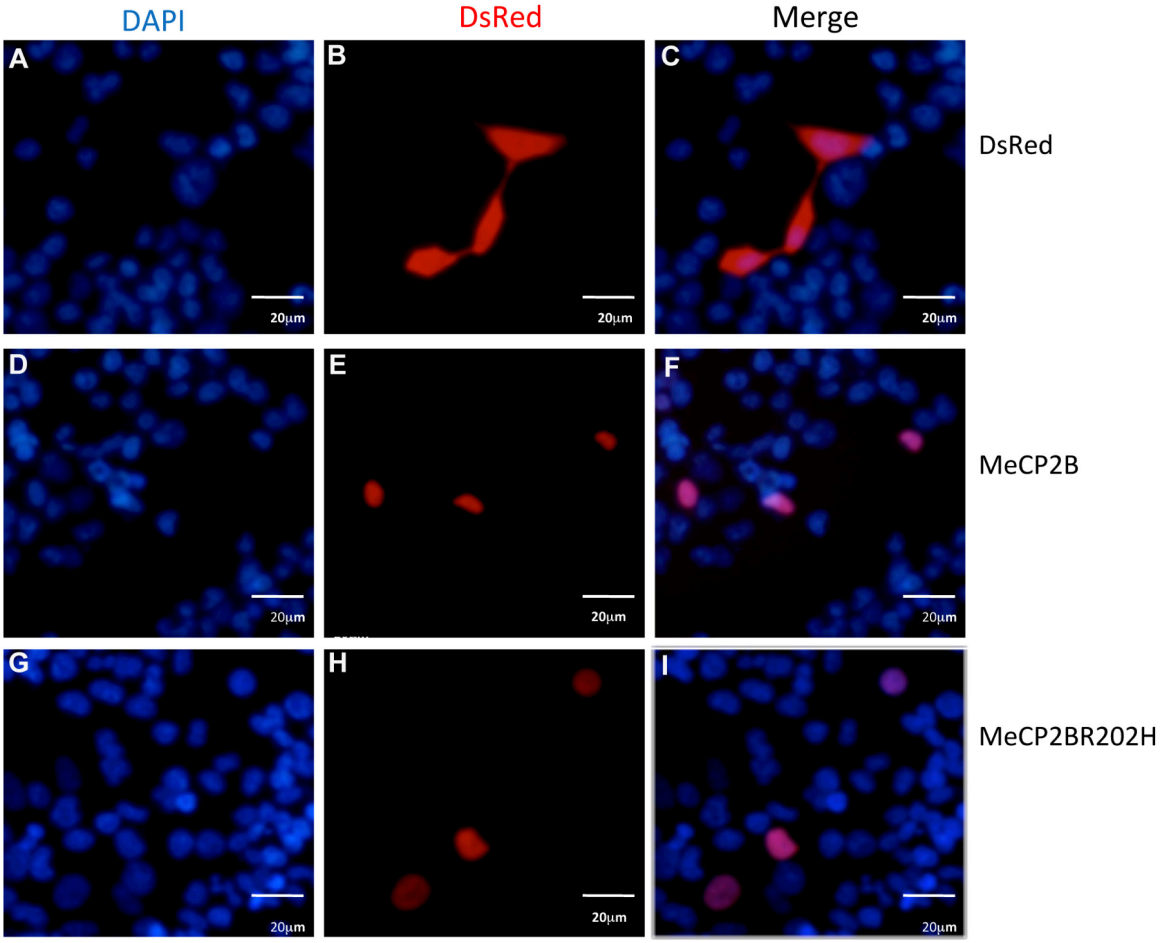

Supplementary Figure 1: MeCP2 R202H mutant proteins located in nuclear as well as wt MeCP2. Plasmids pDsRed-Monomer-N1, pDsRed-Monomer-N1-MECP2B and pDsRed- Monomer-N1-MECP2B R202H were transfected by calcium phosphate method in HEK293 cells. 4% formaldehyde fixed cells on coverslips were mounted in anti-fading agent with DAPI/Vectashield on glass slides. Fluorescence images were taken taken at X400, and the scale bar is 20μm. Blue signals represented DAPI stained nuclei, red signals were DsRed monomer or DsRed fused MeCP2B or MeCP2B R202H, pink signals were blue and red merged signals. DsRed monomer expressed in cell cytosol, DsRed fused MeCP2B or MeCP2B R202H located in nuclei.
